# Management of Incisional Self-Harm of the Upper Limb: A Systematic Review

**DOI:** 10.1016/j.jpra.2023.01.003

**Published:** 2023-02-28

**Authors:** Arun Somanathan, Alice Lee, Conrad J. Harrison, Jeremy N. Rodrigues

**Affiliations:** 1 Oxford University Medical School, University of Oxford, Oxford, UK; 2 Department of Surgery and Cancer, Imperial College London, London, UK; 3 Nuffield Department of Orthopaedics, Rheumatology and Musculoskeletal Sciences, Oxford, UK; 4 Clinical Trials Unit, University of Warwick, Coventry, UK; 5 Department of Plastic Surgery, Stoke Mandeville Hospital, Buckinghamshire Healthcare NHS Trust

**Keywords:** self-harm, hand surgery, outcomes, tendon repair, nerve repair

## Abstract

**Background:**

The incidence of incisional self-harm of the upper limbs is increasing, and recurrence rates are high. It is not known whether different wound treatment strategies (dressings only vs. surgery) or the operative setting (main theatre vs. non-main theatre) affect wound or mental health-related outcomes.

**Methods:**

Four electronic databases (Ovid MEDLINE, OVID EMBASE, PsycINFO and CENTRAL) were searched from inception to 14/09/2021 for studies which describe the management of incisional self-harm wounds of the upper limb(s) in adults and children. Dual-author screening and data extraction were conducted according to the Preferred Reporting Items for Systematic Reviews and Meta-Analyses guidelines.

**Results:**

In total, 19 studies (1477 patients) were included. Overall, the evidence was limited by a paucity of comparative data on wound management strategy and setting, and poor-quality outcome reporting. Only four studies clearly identified the operative setting for definitive wound management (two in main operating theatres, one in the emergency department and one using both settings, depending on injury severity). Few studies inconsistently reported surgical outcomes (n=9) or mental health outcomes (n=4), hindering evidence synthesis.

**Conclusion:**

Further investigation is needed to determine the most cost-effective management strategies and settings for these injuries.

## Introduction

Self-harm can be defined as self-injurious behaviour via various mechanisms (such as self-cutting, poisoning or burning), regardless of intent.^1^ Self-cutting, or incisional self-harm, is the most common form of non-suicidal self-harm, affecting approximately 3% of males and 5% of females in the UK.^2^ Approximately 20% of all self-inflicted wounds are lacerations to the upper limb.^3^ Many self-inflicted wounds are managed surgically in operating theatres, often with multiple procedures.^4^ Self-harm is associated with high readmission rates (up to 50%) due to recurrence^4^ and significantly increased risk of suicide and death from natural causes.^5^ This results in high costs to health systems: £128.6 million for all-cause self-harm presentations to English hospitals in 2013.^6^^,^^7^

Optimising the surgical and psychological treatment of these injuries to prevent recurrence and improve cost-effectiveness is therefore a priority. The recently drafted National Institute for Health and Care Excellence (NICE) guidelines for self-harm (NG10148) emphasise the importance of a psychosocial assessment after every episode of self-harm at the earliest opportunity (National Institute for Health and Care Excellence, 2022)^8^. They also recommend that all patients are offered treatment for the physical consequences of self-harm, regardless of their willingness to accept psychosocial assessment or treatment. However, the practical implementation of these guidelines within hand surgery services can be challenging. The British Society for Surgery of the Hand (BSSH) guidelines for hand trauma make no reference to the self-harm population or their specific needs.^9^ There is also evidence of negative stereotyping of patients who self-harm by healthcare professionals in surgical services^10^^,^^11^; this includes judgemental beliefs regarding motivation for the act (i.e. that it is ‘attention seeking’), restriction of surgical treatment due to perceived futility (due to wound tampering or repeated self-harm) or surgical intervention reinforcing self-harm behaviours.

Decision-making for operative vs. best non-operative care should be discussed with patients on an individual basis.^9^ Current BSSH hand trauma guidelines recommend surgical management in an operating theatre for most structural injuries, except extensor tendons which may be repaired in a procedural room.^9^ However, this may change following the recently published guidelines for operating outside of main theatres, also by the BSSH, which suggest that simple bony and most soft-tissue elective and emergency hand surgery can be conducted safely in non-main theatre sites.^12^ There is limited high-quality data comparing theatre with non-theatre settings, but it is becoming more commonplace, facilitated by wide awake local anaesthesia no tourniquet techniques, with reassuring preliminary outcome and health economic data.^13^, ^14^, ^15^ The alternative is active non-operative management (i.e. wound washout and dressings); this may help in reducing hospital admissions,^16^ but there are no high-quality outcome data comparing operative with non-operative management.

The primary aim of this review is to compare the management of incisional self-harm wounds of the upper limb (operative vs. non-operative, and theatre vs. non-theatre settings), with respect to surgical and mental health outcomes. Our secondary aims include comprehensively describing the epidemiology of incisional self-harm injuries of the upper limb, regarding patient and injury characteristics, psychological and surgical management approaches, and their outcomes.

## Methods

This systematic review adheres to a prespecified protocol and the Preferred Reporting Items for Systematic Reviews and Meta-Analyses (PRISMA) 2020 checklist.^17^ The protocol for this review was registered on PROSPERO (CRD42021282971).

### Study identification

The inclusion and exclusion criteria are shown in Table 1. The following electronic databases were searched from inception to 14/09/2021: Ovid MEDLINE, OVID EMBASE, PsycINFO and CENTRAL. A search string was developed to identify relevant papers, including key search terms and relevant medical subject headings (**Appendix; Table S1**). Studies returned by database searches were compiled and de-duplicated using Covidence© software. Two reviewers (AL and AS) independently screened articles against prespecified inclusion and exclusion criteria in two stages (title and abstract, and full text). Discrepancies between reviewers were resolved through discussion; a third author (JNR) was consulted if consensus was not reached.

**Table 1:** inclusion and exclusion criteria.

Inclusion criteria	Exclusion criteria
Full length, peer-reviewed original studies published from database inception to 14/09/2021	Non-original studies (systematic review and/or meta-analysis, literature reviews); single case studies; or cadaveric, animal or laboratory-based studies
Studies which describe incisional self-harm of the upper limb(s), regardless of suicidal intent, in adults and children	Studies which describe only non-incisional self-harm (e.g, ingestion, burns)
Studies which describe wound management in both traditional theatre and non-theatre environments (ward, emergency department, clinic room or minor operating room)	Studies which describe only incisional self-harm not on the upper limb(s)
	Studies which did not describe wound management
	Non-English-language articles
	Studies of completed suicide

### Data extraction

Two reviewers (AL and AS) independently extracted data using a piloted data extraction form developed for the purpose of the review. This included study details (year and country of publication, average follow-up time), participant demographics (age, sex, psychiatric comorbidities), injury characteristics (anatomical location and laterality, number and type of injured structures) and management approach (principles of wound management and location, and provision of mental health assessment). Information was extracted per injured structure (tendon, nerve, artery) and in commonly encountered groups of structures, i.e. radial triad (median nerve, palmaris longus and flexor carpi radialis injuries) and ulnar triad (ulnar nerve, ulnar artery and flexor carpi ulnaris injuries). Upper limb structures were also classified into superficial, middle and deep anatomical layers, as previously described.^18^ Non-structural injuries were defined as skin-only incisions or lacerations. Non-theatre settings included emergency departments, wards, clinic rooms and minor operating rooms.

Extracted outcomes were mental health related and surgical/functional. Mental health outcomes included further attempted self-harm (any), further suicide attempt and further episode(s) of incisional self-harm. Post-operative outcomes included clinically diagnosed wound infection, any re-operation related to the original injury (including re-repair and tenolysis) and adherence to surgical follow-up. Functional outcomes included any patient-reported outcome measure for the upper limb and any measure of tendon, intrinsic muscle or motor/sensory nerve function.

### Data synthesis

The following were summarised narratively: study details; patient and injury characteristics; management approach (operative vs. non-operative) and setting of operative wound management. Suitability for meta-analysis of surgical and mental health outcomes was determined by author consensus based on the clinical and statistical heterogeneity of the included studies. Quantitative synthesis was not appropriate; hence, narrative synthesis was undertaken following the synthesis without meta-analysis (SwiM) guidance.^19^

### Risk-of-bias assessment

The National Institutes of Health Quality Assessment Tool was used to assess the risk of bias for Observational Cohort and Cross-Sectional Studies.^20^ Two reviewers (AL and AS) independently assessed each included article. Disagreements between reviewers were resolved by discussion; a third reviewer (JNR) was consulted if needed.

## Results

### Study selection

**Figure 1:**
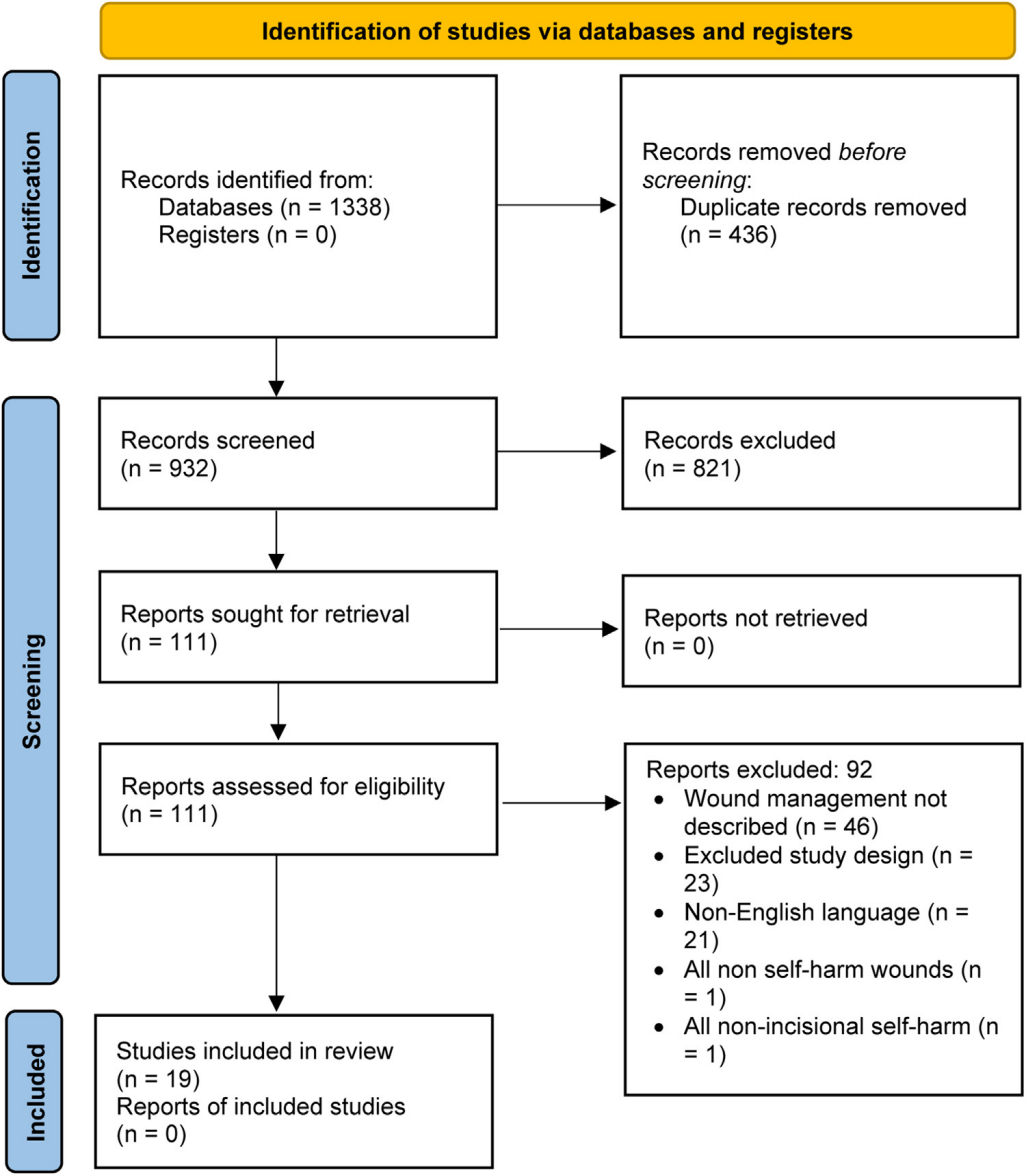
PRISMA flow diagram.

Database searches returned 932 non-duplicate citations, of which 19 were included in the review (Figure 1).

### Study characteristics

A list of included studies is provided in **Table S1**. Most (n=17) were retrospective cohort studies and two were prospective cohort studies. There were no randomised-controlled trials or comparative cohort studies. Eleven studies only included patients with self-inflicted injuries; six also included accidental or non-self-inflicted wounds. Most studies (n=17) aimed to describe the characteristics and/or surgical outcomes of upper limb incisional self-harm injuries. One study specified factors relating to post-closure wound infection as the primary outcome.^21^ Two studies compared injury characteristics of self-harm and accidental injuries.^22^^,^^23^ Others compared injury characteristics of patients with and without suicidal intent,^24^ and with superficial vs. deep wrist wounds.^25^ Two studies aimed to evaluate new interventions, namely, a new institutional suicide prevention plan^21^ and image-guided foreign body removal in the treatment of self-embedded foreign bodies.^26^ Only three studies specified a follow-up time (average range: 6–48 months).

### Risk-of-bias assessment

The overall risk of bias for the included studies was fair (13, 68%), poor (4, 21%) and good (2, 11%) (**Table S2**).

### Patient characteristics

A total of 1477 patients were included; 585 (49%) were male, and the mean age ranged from 16 to 57 years. Some studies specified their population, for example, self-inflicted stab wounds,^27^ flexor zone 5 lacerations,^28^ spaghetti wrist injuries,^29^^,^^30^ upper extremity vascular injuries^22^ and self-embedded foreign bodies.^26^ Where psychiatric comorbidities were described, 325 (35%) patients had depression or low mood, 180 (19%) previous self-harm, 73 (8%) previous suicide attempt(s), 71 (8%) any substance misuse disorder, 61 (6%) bipolar disorder, 45 (5%) schizophrenia or psychotic spectrum disorder, 40 (4%) personality disorder and 11 (1%) anxiety disorder. Where reported, 360 (43%) reported substance use at the time of injury.

### Injury characteristics

Where specified, 356 (82%) patients had unilateral self-inflicted upper limb injuries and 78 (18%) had bilateral injuries. Where injury characteristics were described in detail, 381 (36%) were superficial with no structural injury. A total of 894 structures (tendons, arteries or nerves) were injured, or 0.84 structures per patient (**Table S3**).

### Surgical management and outcomes

Four studies clearly identified the setting for definitive wound management: two in main operating theatres, one in the emergency department and one using both main operating theatre and the emergency department, depending on injury severity. Those studies which specified main operating theatre management included cohorts of flexor tendon zone 5 injuries^28^ and upper limb vascular injuries.^22^ Brudvik et al. specifically studied the infection rates of wounds sutured in a Norwegian emergency department and excluded structural injuries.^31^ Maloney et al. managed wounds with no structural injuries, or extensor or palmaris longus tendon injury only, in the emergency department; other (unspecified) tendon injuries and an ulnar nerve injury were referred for specialist management.^32^ No studies described non-operative management.

Nine studies reported surgical or functional outcomes. No comparisons were made by setting (theatre vs. non-theatre settings). One study reported wound infection in 2/3 self-inflicted wounds closed in the emergency department.^31^ Two studies reported re-operation rates (combined n=5/44, 11%), for scar revision (n=2), tendon transfer for ulnar nerve palsy (n=2) and tenolysis (n=1). Two studies reported patient compliance with surgical follow-up (combined n=138/528, 26%). Dewing et al. reported paraesthesia (23/228, 10%) and chronic pain secondary to neuroma (3/228, 1%); stiffness/tendon rupture/methicillin-resistant Staphylococcus aureus (MRSA) infection were reported as a composite outcome; hence, the data could not be disaggregated.^33^ Four studies reported functional outcomes with respect to tendon, intrinsic muscle and motor/sensory function. Kim et al. described four patients with long-term functional deficits secondary to median and ulnar nerve injuries, but did not detail further.^34^ Functional outcome measures were not uniform across studies: Ersen et al. measured the percentage of full range of motion and/or finger flexion distance from the distal palmar crease, Kapandji index, the Bunnell-Littler test and two-point discrimination,^35^ whereas Gu et al. and Jeong et al. evaluated tendon function according to the Lister classification and motor/sensory function according to Seddon.^36^^,^^37^ For the latter two studies, the combined hand functional grading scores were excellent (94, 52%), good (36, 20%), fair (15, 8%) and poor (13, 7%), and the tendon function grading scores were excellent (94, 52%), good (40, 22%), fair (15, 8%) and poor (9, 5%). None of the studies included patient-reported outcome measures.

### Mental health management and outcomes

Seven studies described psychiatric management. Those that did offered same-day psychosocial assessment to 296 (76%) patients and follow-up to 102 (26%) patients. Four studies reported mental health outcomes within the study period. One study reported further attempted or completed suicide (5, 4%), and three studies reported further instances of self-harm (13, 6%). One study described ‘several’ repeated incisional self-harm episodes but did not quantify this further.^33^ Jeong et al. reported 13 post-discharge hospitalisations for ‘psychological problems’.^37^

## Discussion

Our key finding is that there is a paucity of comparative data for different management approaches (operative vs. non-operative, and theatre vs. non-theatre settings) for incisional self-harm injuries of the upper limb and underreporting of mental health assessment and outcomes.

We were unable to compare theatre and non-theatre settings for wound management (our primary outcome) because most studies did not describe the operative setting. However, a notable proportion of the injury patterns described in the included studies would not necessarily need formal surgical exploration and repair (e.g. superficial only, or involving potentially expendable structures such as palmaris longus). If these patterns are generalisable, then non-operative or non-theatre management might be appropriate in some circumstances. This is an area that may merit further investigation. It is unclear whether wound management approach or setting affects functional outcomes, recurrent self-harm risk or other mental health outcomes. Inconsistent surgical, functional and mental health outcome reporting hindered evidence synthesis and could be standardised. This may be partly facilitated by a core outcome set for flexor tendon injuries (currently in development)^38^ but the broader well-being of these patients must also be considered in future efforts to standardise measurement.

The proportion of patients offered psychosocial assessment during admission and follow-up was short of NICE standards and was likely overestimated given that two-thirds of included studies did not describe psychiatric management. Few studies reported rates of further attempted or completed suicide and recurrent self-harm within the study period. Where reported, events were rare. However, the reported figures may be underestimates due to underreporting, given repetition rates of up to 50% in the non-surgical literature^39^^,^^40^; these studies were not included in this review because they did not describe wound management. Consequently, we currently have limited evidence available on which to base holistic assessment and treatment pathways in this area.

Patients with incisional self-harm injuries have the highest recurrence rates,^40^ yet are significantly less likely to have a psychosocial assessment compared with other methods, e.g. self-poisoning.^39^ This is a missed opportunity, given recent evidence demonstrating significantly reduced self-harm recurrence with psychosocial interventions.^41^^,^^42^ All healthcare professionals (including hand surgeons) should ensure that patients under their care have a psychosocial assessment by a suitable professional and aftercare arranged prior to hospital discharge, as suggested by new (draft) NICE guidance.^8^ Improved quality of reporting on psychological outcomes in the hand surgery literature is a priority. There is no core outcome set for self-harm injuries, but one for discharge from inpatient mental health services recommends that at least the following are included: readmission, completed suicide, service user-reported psychological distress and quality of life.^43^

The near-equal gender distribution in this study may be explained by men being more likely to use sharp objects in non-fatal suicide attempts,^44^ although all-cause self-harm is significantly more common amongst female patients.^45^ The high incidence of self-harm in patients with psychiatric comorbidities, particularly mood disorders and substance misuse, has been reported previously^46^, ^47^, ^48^, ^49^ and reiterates the importance of screening for psychiatric symptoms or diagnoses at presentation, and ensuring effective multidisciplinary care.^50^

The strengths of this review include the comprehensive search of four electronic databases and dual-author screening and data extraction, according to PRISMA methodology.^17^ Limitations include the exclusion of non-English-language articles and inability to use data due to varying classifications of structural injury (e.g. different definitions of what constituted a superficial vs. deep injury). Most studies were retrospective cohorts with small participant numbers. Some studies specifically excluded structural injuries^34^ or superficial injuries,^23^ or only included specific injury patterns (e.g. vascular injuries),^22^ which may have biased the injury characteristics.

## Conclusions

Incisional self-harm of the upper limb is prevalent in both genders with high rates of psychiatric comorbidity. There is a paucity of comparative data on different treatment approaches (operative vs. non-operative) and settings for wound management (theatre vs. non-theatre), and the effect (if any) on wound-related outcomes, mental health-related outcomes and health economic outcomes; this warrants further investigation. Few studies reported surgical, and particularly mental health, outcomes, with inconsistent outcome measures, hindering evidence synthesis. High-quality comparative studies with standardised outcome reporting are warranted. All healthcare professionals have a responsibility to ensure an appropriate psychosocial assessment is performed prior to discharge.

## Funding statement

Conrad J. Harrison is funded by a National Institute for Health Research (NIHR) Doctoral Research Fellowship (NIHR300684) for this research project. Jeremy N. Rodrigues is funded by an NIHR postdoctoral fellowship (PDF-2017-10-075). This document presents independent research funded by the NIHR. The views expressed are those of the authors and not necessarily those of the NHS, the NIHR or the Department of Health and Social Care.

## Ethical approval

Not required.

## Conflict of interest statement

None declared.

## Appendix Supplementary materials

Image, application 1
