# Supplementary material for: Management of Incisional Self-Harm of the Upper Limb: A Systematic Review
Source: JPRAS Open. 2023 Feb 28;36:76–84. doi: 10.1016/j.jpra.2023.01.003 (PMC10184042; doi:10.1016/j.jpra.2023.01.003)
Supplement: Supplementary file 1 [file mmc1.docx]

**Appendix**

**Table S1.** Database search string.

| 1 | exp Upper Extremity/ |
| --- | --- |
| 2 | exp Hand Injuries/ |
| 3 | exp Arm Injuries/ |
| 4 | (forearm* or wrist* or hand or hands or finger* or thumb* or arm or arms or "upper limb*" or elbow* or palm or palms).ti,ab. |
| 5 | self-injurious behavior/ or exp self mutilation/ or suicide/ or exp suicidal ideation/ or exp suicide, attempted/ or exp suicide, completed/ |
| 6 | (self adj4 harm*).ti,ab. |
| 7 | (self adj4 injur*).ti,ab. |
| 8 | parasuicid*.ti,ab. |
| 9 | (self adj4 inflict*).ti,ab. |
| 10 | (self adj4 mutilat*).ti,ab. |
| 11 | (selfdestruct* or selfinflict* or selfinjur* or selfmutilat* or automutilat*).ti,ab. |
| 12 | (self adj4 destruct*).ti,ab. |
| 13 | (auto adj mutilat*).ti,ab. |
| 14 | (self adj4 (cut or cuts or cutting or cutter)).ti,ab. |
| 15 | suicid*.ti,ab. |
| 16 | 5 or 6 or 7 or 8 or 9 or 10 or 11 or 12 or 13 or 14 or 15 |
| 17 | 1 or 2 or 3 or 4 |
| 18 | 16 and 17 |
| 19 | exp Wounds, Penetrating/ |
| 20 | exp Lacerations/ |
| 21 | (lacerat* or sharp* or cut or cuts or cutter or cutters or cutting or cuttings or penetrat* or incis* or stab or stabs or stabbed or stabbing or stabbings).ti,ab. |
| 22 | 19 or 20 or 21 |
| 23 | 18 and 22 |
| 24 | remove duplicates from 23 |

**Table S2.** Risk-of-bias assessment.

| **First author** | **Year** | **Q1** | **Q2** | **Q3** | **Q4** | **Q5** | **Q6** | **Q7** | **Q8** | **Q9** | **Q10** | **Q11** | **Q12** | **Q13** | **Q14** | **Overall rating** |
| --- | --- | --- | --- | --- | --- | --- | --- | --- | --- | --- | --- | --- | --- | --- | --- | --- |
| Cho | 2020 | Yes | Yes | Yes | Yes | No | No | N/A | N/A | N/A | N/A | Yes | CD | N/A | Yes | Fair |
| Brudvik | 2015 | Yes | Yes | Yes | Yes | No | Yes | Yes | Yes | Yes | N/A | Yes | CD | No | Yes | Fair |
| Bukur | 2011 | Yes | Yes | Yes | No | Yes | No | Yes | Yes | Yes | N/A | Yes | CD | CD | Yes | Fair |
| Chuinard | 1979 | Yes | Yes | Yes | Yes | No | No | Yes | Yes | Yes | N/A | Yes | CD | N/A | No | Fair |
| Dewing | 2010 | No | No | Yes | Yes | No | No | Yes | Yes | CD | N/A | No | CD | Yes | No | Poor |
| Ersen | 2017 | Yes | Yes | CD | Yes | No | No | Yes | Yes | Yes | N/A | Yes | CD | No | No | Fair |
| Fujioka | 2012 | Yes | Yes | CD | Yes | Yes | No | Yes | Yes | Yes | N/A | Yes | CD | Yes | Yes | Fair |
| Gu | 2012 | No | Yes | CD | No | NR | No | Yes | Yes | Yes | N/A | No | CD | Yes | Yes | Fair |
| Jeong | 2020 | Yes | Yes | CD | No | No | No | Yes | Yes | Yes | N/A | No | CD | CD | Yes | Fair |
| Kim | 2021 | Yes | Yes | CD | Yes | Yes | Yes | Yes | Yes | Yes | N/A | No | CD | Yes | No | Good |
| Kisch | 2019 | Yes | Yes | CD | Yes | No | Yes | Yes | Yes | Yes | N/A | Yes | CD | CD | CD | Fair |
| Lee | 2016 | Yes | No | Yes | CD | Yes | Yes | Yes | Yes | Yes | N/A | Yes | CD | CD | CD | Fair |
| Maloney | 1987 | No | No | CD | No | No | CD | Yes | Yes | CD | N/A | CD | CD | CD | No | Poor |
| Park | 2020 | Yes | Yes | CD | No | No | CD | Yes | Yes | Yes | N/A | Yes | CD | CD | No | Fair |
| Raza | 2014 | Yes | Yes | Yes | Yes | No | CD | Yes | No | Yes | N/A | Yes | CD | CD | No | Good |
| Topal | 2010 | Yes | No | Yes | Yes | No | CD | Yes | No | Yes | N/A | Yes | CD | CD | No | Fair |
| Vaughn | 2016 | Yes | Yes | Yes | Yes | No | CD | Yes | No | Yes | N/A | No | CD | No | No | Poor |
| Weinzweig | 1998 | Yes | No | CD | Yes | No | CD | Yes | No | Yes | N/A | No | CD | No | No | Poor |
| Young | 2010 | Yes | No | N/A | Yes | No | CD | Yes | No | CD | N/A | CD | CD | CD | No | Fair |

Abbreviations: CD, cannot determine; NR, not reported.

**Table S3**. Summary of structural injuries for included patients.

| **Structural injury** | | **N (%)** |
| --- | --- | --- |
| Tendons | PL | 186 (18) |
|  | FCR | 133 (13) |
|  | FCU | 101 (10) |
|  | FDS | 123 (12) |
|  | FDP | 35 (3) |
|  | FPL | 29 (3) |
|  | Extensor tendon | 15 (1) |
| Nerves | Ulnar nerve | 97 (9) |
|  | Median nerve | 137 (13) |
|  | SBRN | 6 (1) |
|  | Radial nerve | 1 (0) |
|  | >1 nerve | 15 (1) |
| Arteries | Ulnar artery | 123 (12) |
|  | Radial artery | 100 (9) |
|  | Other arterial injury | 34 (3) |
|  | >1 artery | 12 (1) |
| Radial triad | Median nerve + PL + FCR | 30 (3) |
| Ulnar triad | Ulnar nerve + ulnar artery + FCU | 14 (1) |
| Superficial layer^a^ | PL, FCR, FDS 3, FDS 4, FCU | 165 (26) |
| Middle layer^a^ | Ulnar nerve, ulnar artery, FDS 2, FDS 5, median nerve, radial artery | 145 (23) |
| Deep layer^a^ | FPL, FDP | 106 (17) |

^a^Only 7 studies presented data which could be aggregated into superficial, middle and deep layers. FCR, flexor carpi radialis; FCU, flexor carpi ulnaris; FDP, flexor digitorum profundus; FDS, flexor digitorum superficialis; FPL, flexor pollicis longus; PL, palmaris longus; SBRN, superficial branch of radial nerve.
